# Supplementary material for: Assemblage of Focal Species Recognizers—AFSR: A technique for decreasing false indications of presence from acoustic automatic identification in a multiple species context
Source: PLoS One. 2019 Dec 5;14(12):e0212727. doi: 10.1371/journal.pone.0212727 (PMC6894755; doi:10.1371/journal.pone.0212727)
Supplement: S1 Supporting Information — (PDF) [file pone.0212727.s001.pdf]

S1 Supporting Information

Preliminary Recognizers - General Modelling approach

We created a sequence of four recognizers, each with a training and testing stage. These four recognizers were divided in two groups, preliminary recognizers and optimised recognizers. The preliminary recognizers are not described in the main text and are only presented here in the supporting information. The preliminary recognizers, a) *Initial Recognizer* and b) *Edited Recognizer*, were built from training files extracted from active recordings. They were tested against a 2.5 minute long sound file containing a subset of the training calls.

The optimised recognizers, c) *Multispecies Recognizer* and d) *AFSR*, received additional training calls extracted from passive acoustic recordings (as described in the paper). The process for selecting these was fast-tracked by running the b) *Edited Recognizer* over these PAM recordings as this provided an initial identification that could then be confirmed by a seabird expert. The optimised recognizers were tested more rigorously against a 10 minute long sound file created from fragments of the PAM recordings.

The two preliminary recognizers were crucial for the construction of the sound file training data set (total 179 MB) used in the paper. Since the a) *Initial Recognizer* and b) *Edited Recognizer* are not described in the paper, a short description of these two recognizers is given in this Appendix.

Description of the Preliminary Recognizers

a) Initial Recognizer

Manually annotated label files were created for all sound files initially selected from the active recordings previously described. The inclusion of these files into the set of training files was performed until the recognizers were able to identify calls from all the five petrels, generating a single recognizer containing all the species. This initial selection of sound files and manual creation of the annotation text files generated 189 MB of training files.

b) Edited Recognizer

Poor quality training files used for a) *Initial Recognizer* were identified by visual and aural inspection of spectrograms and excluded to build the training files set of the *Edited Recognizer*. Moreover, additional

good quality files were identified from the active recordings and added. The minimal interval between two calls or different sound categories was here defined as 0.1 second. It means that two consecutive calls from the same species would be separately annotated if the interval between them was longer than or equal to 0.1 second. This avoided inclusion of unnecessary background sound within sound fragments used to create the recogniser statistical profile for each category during the training stage. After the edits, file exclusions, and additions, the resulting *Edited Recognizer* had 109 MB of training sound files.

The Edited Recognizer was then ran over the PAM recordings to fast track finding more examples of calls. All these calls extracted from the active and PAM recordings were combined into a single set of training sound files, which were then used for both our multispecies recogniser and our AFSR (assemblage of focal species recognizers). An overall modelling workflow diagram including the preliminary models (*Initial Recognizer* and *Edited Recognizer*) and optimized models (*Multispecies Recognizer* and *AFSR*) is presented in the Fig A.

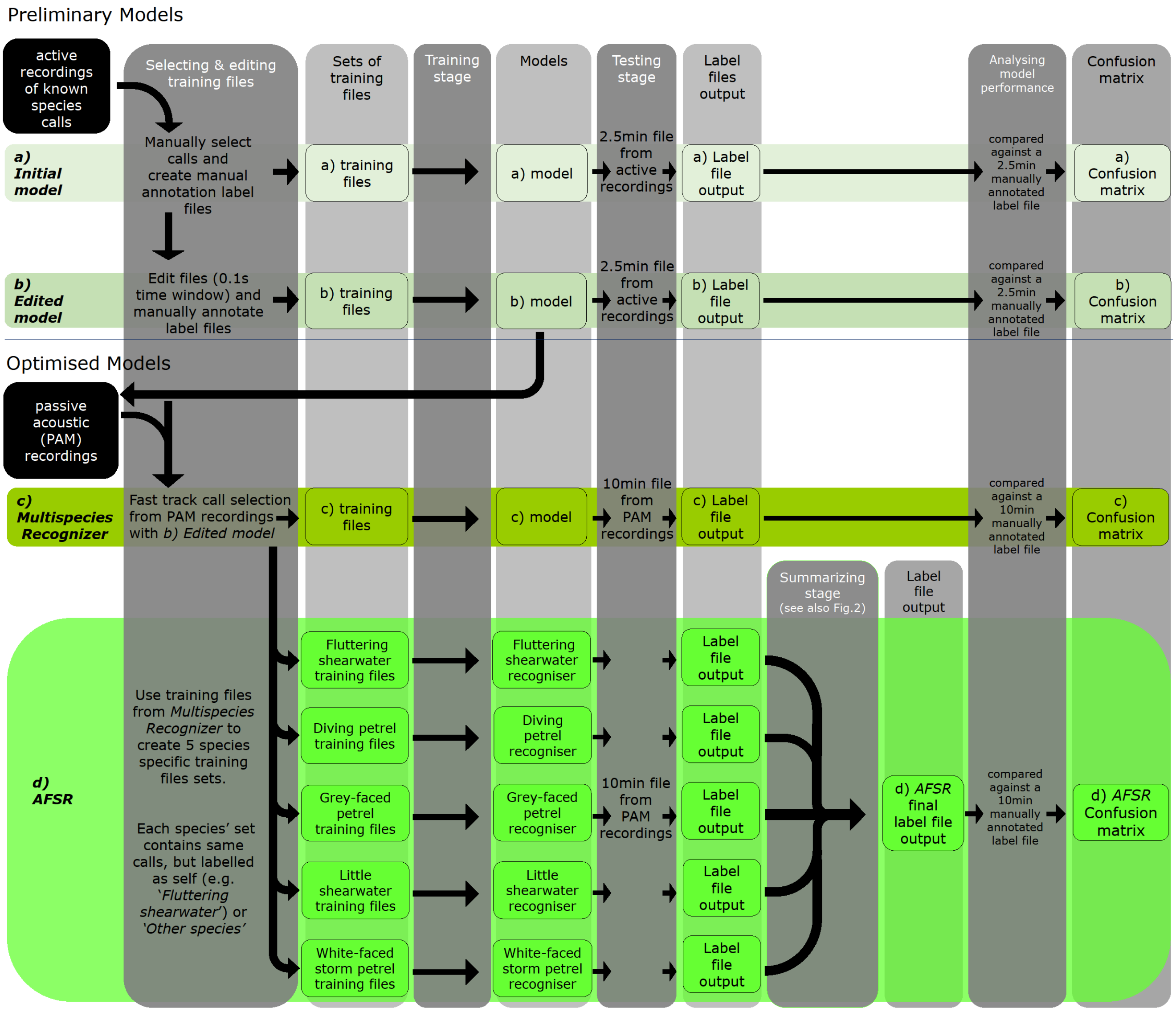

Fig A. Overall Modelling Workflow diagram including Preliminary and Optimized Models Each modelling approach is represented in horizontal lines. The workflow within each approach runs from left to right (columns) and the workflow from one modelling approach to the next runs from top to bottom.

Preliminary Recognizers' Results

When compared with the 2.5 minute manually annotated file the *a) Initial Recognizer* achieved 63% similarity, while the *b) Edited Recognizer* achieved 69%. The *Initial Recognizer's* results for Fluttering shearwater, Common diving petrel, and Grey-faced petrel were considered poor due to the high false positives (43%, 50%, and 94%) and low precision (57%, 50%, and 6%, respectively) achieved. Overall the *Edited Recognizer* was only slightly more accurate than the *Initial Recognizer*, showing lower false positive rates and higher precision for Grey-faced petrel, Fluttering shearwater and White-faced storm petrel (Table 1). However, there was an increase in the false positive rate (from 4% in the *Initial recognizer* to 17%) for Little shearwater, and extremely poor results for Common diving petrel, with no true positive indication of presence (false positive rate of 100%).

The preliminary recognizers did not achieve a high performance and represent only a previous step for building the optimised recognizers, c) *Multispecies Recognizer* and d) *AFSR*.

Table 1. Total false positive rate and Precision per species achieved by each of the four recognizers tested

Species	a) Initial Recognizer		b) Edited Recognizer		c) Multispecies Recognizer		d) AFSR	
	Total false positive rate	Precision	Total false positive rate	Precision	Total false positive rate	Precision	Total false positive rate	Precision
Common diving petrel	0.5	*0.5*	1	*0*	0.09	*0.88*	0.01	*0.97*
Grey-faced petrel	0.94	*0.06*	0.06	*0.94*	0.1	*0.88*	0.05	*0.92*
Little shearwater	0.04	*0.96*	0.17	*0.83*	0.05	*0.93*	0	*1*
Fluttering shearwater	0.43	*0.57*	0.04	*0.96*	0.01	*0.99*	0	*1*
White-faced storm petrel	0.04	*0.96*	0	*1*	0.02	*0.98*	0	*1*

Total false positive rates (underlined) and precision (*italic*) for each species were calculated from the values generated by confusion matrices for each recognizer and are presented here in a scale from 0 to 1, being 1 equals to 100%.
