# Supplementary material for: Assemblage of Focal Species Recognizers—AFSR: A technique for decreasing false indications of presence from acoustic automatic identification in a multiple species context
Source: PLoS One. 2019 Dec 5;14(12):e0212727. doi: 10.1371/journal.pone.0212727 (PMC6894755; doi:10.1371/journal.pone.0212727)
Supplement: S2 Supporting Information — (PDF) [file pone.0212727.s002.pdf]

S2 Supporting Information

DATA ACCESSIBILITY

The MatlabHTK package can be downloaded from:

https://github.com/LouisRankard/matlabHTK

The use of MatlabHTK requires the beforehand installation of Octave (Eaton et al., 2014) and HTK (HTK, Young et al., 2016).

The AFSR_summarizing script can be downloaded from:

https://github.com/klee8/AFSR.

Data deposited in the Dryad Repository: http://dx.doi.org/10.5061/dryad.6kv00.
