# Supplementary material for: Assemblage of Focal Species Recognizers—AFSR: A technique for decreasing false indications of presence from acoustic automatic identification in a multiple species context
Source: PLoS One. 2019 Dec 5;14(12):e0212727. doi: 10.1371/journal.pone.0212727 (PMC6894755; doi:10.1371/journal.pone.0212727)
Supplement: S3 Supporting Information — (PDF) [file pone.0212727.s003.pdf]

S3 Supporting Information

AFSR SUMMARIZING RULES

A set of rules were used to summarize the five annotation outputs into one annotation. A script called AFSR_summarizing (https://github.com/klee8/AFSR**)** was created for summarizing the five annotation text files (label format) into one, following seven rules:

1. When the five independent species-specific recognisers indicate the same category, the indicated category should be used. For example, if all five indicate "*Background*", then "*Background*" should be used in the summarized annotation output. If all five indicate "*Other Species*", then "*Other Species*" should be used in the summarized annotation output.

2. The "*Background*" category should be used in the summarized annotation output if it appears in at least one of the independent species-specific recognisers' outputs. There are only two exceptions: i) when "*Background*" appears at the same time as "*Noise*", the category "*Noise*" should be used in the summarized annotation output, or ii) if among the five recognisers annotation outputs there are four different categories indicated, the category "*Other Species*" should be used in the summarized annotation output (even when one of the four categories indicated is "*Background*").
3. The "*Noise*" category should be used every time it appears in one of the independent species specific recognisers.

4. If two categories are indicated and one of the categories is one of the target species and the other is "*Other Species*", use the target species category. For example, if one recogniser indicates "*Grey-faced Petrel*" and all the other four recognisers indicate "*Other Species*", "*Grey-faced Petrel*" should be used in the summarized annotation output.

5. If three categories are indicated, the category "*Other Species*" should be used in the summarized annotation output. For example, if two of the categories indicated are target species and the other is "*Other Species*", "*Other Species*" should be used in the summarized annotation output. The exception is when three categories are indicated and one of them is "*Background*", then "*Background*" should be used in the summarized annotation output.

6. If four categories are indicated, "*Other Species*" should be used in the summarized annotation output.

7. After the summarizing process is completed, all fragments indicated as "*Other Species*" in the summarized annotation output should be replaced by "*Unidentified".*

P.S. The "*Noise"* category included sounds typically produced when something touches the microphone during the sound sampling. The "*Noise"* category was not present neither in the 2.5 minute sound file used to test the models *Initial model* and *Edited model* (a and b, respectively), nor in the 10 minute sound file used to test the models *Combined model* (c) and the *Assemblage of Focal Species Recognizers– AFSR* (d). Therefore, this sound category was not mentioned in the paper's body.
