# Supplementary material for: Assemblage of Focal Species Recognizers—AFSR: A technique for decreasing false indications of presence from acoustic automatic identification in a multiple species context
Source: PLoS One. 2019 Dec 5;14(12):e0212727. doi: 10.1371/journal.pone.0212727 (PMC6894755; doi:10.1371/journal.pone.0212727)
Supplement: S4 Supporting Information — (PDF) [file pone.0212727.s004.pdf]

S4 Supporting Information

Confusion Matrix comparing manual species identification *Versus* the Multispecies Recognizer output for a 10 minute long sound file

Manually Annotated	Multispecies Recognizer						
	**Background**	**Common diving petrel**	**Grey-faced petrel**	**Little shearwater**	**Fluttering shearwater**	**White-faced storm petrel**	**Total false positive rate**
**Background**	*0.72*	0.09	0.02	0.04	0.03	0.1	<u>*0.28*</u>
**Common diving petrel**	*0.25*	<u>0.66</u>	0.02	0	0.06	0.01	<u>*0.09*</u>
**Grey-faced petrel**	*0.15*	0.05	<u>0.75</u>	0.01	0.04	0	<u>*0.1*</u>
**Little shearwater**	*0.32*	0	0	<u>0.63</u>	0.05	0	<u>*0.05*</u>
**Fluttering shearwater**	*0.1*	0.01	0	0	<u>0.89</u>	0	<u>*0.01*</u>
**White-faced storm petrel**	*0.12*	0	0	0	0.02	<u>0.86</u>	<u>*0.02*</u>

The proportion of the time in which each category indicated at the manually annotated text file is assigned to each one of the categories at the Multispecies Recognizer's output text file is presented in a scale from 0 to 1 (being 1 equals to 100%) as follows: cells with *values in italic*: negative indications of presence; <u>underlined values</u>**:** true positive indication of presence, values with no special formatting: false positive indication of presence; <u>*values underlined and italic:*</u> total false positive rate for each one of the categories (sum of the cells with no special formatting in each line).
